# Conformational B-Cell Epitope Prediction on Antigen Protein Structures: A Review of Current Algorithms and Comparison with Common Binding Site Prediction Methods

**DOI:** 10.1371/journal.pone.0062249

**Published:** 2013-04-19

**Authors:** Bo Yao, Dandan Zheng, Shide Liang, Chi Zhang

**Affiliations:** 1 School of Biological Sciences, Center for Plant Science and Innovation, University of Nebraska, Lincoln, Nebraska, United States of America; 2 Department of Radiation Oncology, University of Nebraska Medical Center, Omaha, Nebraska, United States of America; 3 Systems Immunology Lab, Immunology Frontier Research Center, Osaka University, Suita, Osaka, Japan; Northwestern University Feinberg School of Medicine, United States of America

## Abstract

Accurate prediction of B-cell antigenic epitopes is important for immunologic research and medical applications, but compared with other bioinformatic problems, antigenic epitope prediction is more challenging because of the extreme variability of antigenic epitopes, where the paratope on the antibody binds specifically to a given epitope with high precision. In spite of the continuing efforts in the past decade, the problem remains unsolved and therefore still attracts a lot of attention from bioinformaticists. Recently, several discontinuous epitope prediction servers became available, and it is intriguing to review all existing methods and evaluate their performances on the same benchmark. In addition, these methods are also compared against common binding site prediction algorithms, since they have been frequently used as substitutes in the absence of good epitope prediction methods.

## Introduction

Antigenic epitopes are regions of the antigen protein surface that are preferentially recognized by antibodies. Prediction of B-cell antigenic epitopes is of direct help to the design of vaccine components and immuno-diagnostic reagents. Usually, B-cell antigenic epitopes are classified as either continuous or discontinuous. The majority of available epitope prediction methods focus on continuous epitopes [1], [2], [3], [4], [5], [6], [7], [8], [9], [10], [11], [12].

On the other hand, discontinuous epitopes dominate most antigenic epitope families [13]. Unfortunately, due to computational complexity and the limited number of known antibody-antigen complex structures, only a limited number of prediction methods exist for discontinuous epitope prediction: CEP [14], DiscoTope [15], BEpro(PEPITO) [16], ElliPro [17], SEPPA [18], EPITOPIA [19], [20] and EPCES [21], EPSVR [22], EPMeta [22], and Bpredictor [23]. Since currently all discontinuous epitope prediction methods require the three-dimensional (3D) structures of antigenic proteins, the small number of available antigen-antibody complex structures greatly limits the development of reliable discontinuous epitope prediction methods. In addition, an unbiased benchmark set is very much in demand [21], [24].

## Results

### Performance of Structure-based Prediction Methods

In the review, we will discuss and evaluate conformational epitope predictors of DiscoTope [15], BEpro(PEPITO) [16], ElliPro [17], SEPPA [18], EPITOPIA [19], [20] and EPCES [21], EPSVR [22], Bpredictor [23], and EPMeta [22] for all of which there exist web servers or free downloadable software packages. DiscoTope [15] integrates with linear combination two scores, the hydrophilicity scale and the epitope log-odds ratios, the latter of which is also one kind of epitopic residue propensity score. BEpro(PEPITO) [16] also applies linear combination to two scores: the epitopic residue propensity and the half sphere exposure values at multiple distances. ElliPro [17] uses only one single score, i.e. residue protrusion index (PI). SEPPA [18] employs the epitopic residue propensity and the compactness of the neighboring residues around one residue (contact number or flat surface), again using linear combination. EPITOPIA [19], [20] applies a naive Bayesian classifier to forty-four physico-chemical and structural–geometrical attributes, including secondary structure, propensity, conservation, solvent accessible surface, and hydrophilicity etc. EPCES [21] devises a special linear method, using a voting mechanism for consensus, to integrate six scores, namely propensity, amino acid side-chain energy value, secondary structure composition, contact number, conservation score, and surface planarity score. One step forward, EPSVR [22] uses the same attributes as EPCES [21] but Support Vector Regression (SVR) to integrate all scores. Bpredictor [23] employs the random forest classifier to adjacent residue distance score, accessible surface area, conservation, secondary structure, and propensity etc. EPMeta is a meta server, which combines EPSVR, EPCES, EPITOPIA, SEPPA, PEPITO, and Discotope1.2.

In general, the features used by these predictors include conservation score, structural features such as secondary composition, geometry characteristics such as protrusion index and planarity score, and amino acid features such as hydrophilicity and propensity (odd-ratios). These attributes can be integrated by linear combination or machine-learning algorithms, such as naive Bayesian classifiers, SVR, and random forest classifiers. Different number of features can be used in a given predictor, from two scores to forty-four attributes. For small numbers of attributes, a simple linear combination can usually work well, whereas large numbers of features often require sophisticated machine-learning algorithms to optimally integrate the scores. Notably, some of these features may be mutual-exclusive or overlapped. For example, the antigenic epitope is frequently located at either a protruding region or a flat surface. In such cases, linearly combining two incompatible terms contradicts the physical basis and will only degrade the performance of a predictor.

The above epitope predictors are trained with most or all of the available antigen-antibody complex structures obtained from x-ray diffraction on crystallized proteins. Therefore, the independent test set compiled by Liang *et al.* [22], which contains 19 protein monomer structures with epitope information derived from experimental methods other than crystal structures, was applied to all methods as an independent evaluation. Table 1 shows the area under receiver operating characteristic curve (AUC) values of all methods. A receiver operating characteristic (ROC) curve represents a dependency of sensitivity and (1-specificity), which is plotted with true positives rate versus false positive rate at various threshold settings. To change the threshold setting, the number of predicted residues is increased in steps of 1% of total surface residues. The mean AUC values are calculated using the method described by Liang *et al.* [22], except for Bpredictor. For Bpredictor, the AUC value is directly obtained from the manuscript, where the same benchmark by Liang et al. was applied as in the current work. Among single servers, EPSVR and Bpredictor have the best performance according to the AUC values. Although EPSVR has the highest mean AUC value, the differences between EPSVR and other servers are not statistically significant (*p-value* >0.05), according to the pairwise t-student tests. The meta server, EPMeta, achieves a mean AUC value of 0.638, which is significantly higher than all single servers.

**Table 1:** List of the conformational B-cell epitope prediction methods and their obtained AUC results.

Method	URL of web server	AUC	Accuracy^b^(%)
DiscoTope [15]	http://www.cbs.dtu.dk/services/DiscoTope/	0.567	15.5
BEpro(PEPITO) [16]	http://pepito.proteomics.ics.uci.edu/	0.570	17.0
ElliPro [17]	http://tools.immuneepitope.org/tools/ElliPro/iedb_input	0.585	14.3
SEPPA [18]	http://lifecenter.sgst.cn/seppa/index.php	0.576	17.2
EPITOPIA [19], [20]	http://epitopia.tau.ac.il/index.html	0.579	17.8
EPCES [21]	http://sysbio.unl.edu/EPCES/	0.586	18.8
EPSVR [22]	http://sysbio.unl.edu/EPSVR/	0.597	24.7
Bpredictor [23]	http://code.google.com/p/my-project-bpredictor/downloads/list	0.598^a^	24.0^c^
EPMeta [22]	http://sysbio.unl.edu/EPMeta/	0.638	25.6

a The AUC value is obtained from the Reference [23].

b 10% of surface residues are returned as predicted epitopic residues.

c Estimated based on the Figure 4 in the Reference [23].

The accuracy, *i.e.* positive prediction rate, is useful for experimental testing. If each server returns 10% of surface residues as predicted epitopic residues, the accuracy is 14.3%, 15.5%, 17.0%, 17.2%, 17.8%, 18.8%, 24.7%, and 25.6% for ElliPro [17], DiscoTope1.2 [15], BEpro (PEPITO) [16], SEPPA [18], EPCES [21], EPITOPIA [19], [20], EPSVR [22], and EPMeta [22] respectively. The accuracy is around 24% for Bpredictor based on Figure 4 in the Reference[23]. The rationale of selecting 10% is because the average length of antigen proteins is around 200 amino acids, and the average size of epitopic patch is about 20 amino acid residues. The current level of accuracy of all predictors is not yet satisfactory. Even the highest accuracy, 25.6% achieved by EPMeta, leaves room for further improvement. If 3% of surface residues are returned as predicted epitopic residues, the accuracy of EPMeta is 31.6%, which is the overall highest value by all conditions and methods.

### Single Chain or Multiple Chains

The recognition of antibody to antigenic epitopes has high specificity; the epitopic surface is not as conserved as other functional protein binding sites, which comes from the conserved functions of protein-protein interactions during evolution. The interfaces of regular protein-protein binding are usually more conserved and have more hydrophobic amino acid residues than non-binding protein surfaces. This makes the exposed protein-protein interfaces relatively easy to distinguish from both the antigenic epitopes and non-binding protein surfaces. In other words, the prediction task for a single chain protein that has both protein-protein binding interfaces and an antigenic epitope is easier than that of a complete protein complex.

In the benchmark, six of the proteins (PDB IDs: 1eku, 1av1, 1al2, 1jeq, 2gib, and 1qgt) possess multiple chains. Therefore, in the evaluation all methods are tested with two different scenarios for these six proteins: prediction on a single chain, where the experimental antigenic epitope is located, and prediction on the whole protein, including all chains. When using multiple chains, all chains are considered, and the total number of surface residues is counted for the intact complex structure. As a result, some methods, such as EPSVR, show dampened performances if the whole protein is used for prediction, resulting in lower mean AUC values for the 6 proteins as compared with predicting based on the single chain containing the antigenic epitope. Therefore, in the future, if sufficient data exist, variant test datasets shall be compiled for different cases, *i.e.* single chain antigens, single chains from antigen complexes, and antigen complexes. A good antigenic epitope predictor shall have satisfying performance on all types of benchmarks.

### Protein Binding Site Prediction Methods

Protein binding site prediction methods are frequently borrowed for conformational epitope prediction [24], [25], since epitopic patches can be considered as one kind of protein binding sites, and due to the lack of many epitope prediction methods for analysis and comparison. The methodologies used by protein binding site prediction and epitope prediction are similar; both integrate some amino acid scoring functions with a machine learning algorithm or other platform to train a prediction model on known data. The major difference is their distinct training sets; while protein binding site prediction uses all known protein-protein binding complexes, an epitope prediction method is trained with antibody-antigen complexes only. Therefore, we also applied the independent benchmark of epitopes to some binding site prediction methods. For this we selected binding site prediction methods that have both demonstrated good performance and convenient web servers for public use. The AUCs achieved by these methods for the epitope benchmark are shown in Table 2. One can see that the performances of the binding site prediction methods to predict B-cell epitopes are significantly lower than all conformational epitope prediction methods. This is not surprising, because all binding site prediction methods are designed based on the conservation and hydrophobicity of binding patches, but B-cell epitopic patches are neither conserved nor more hydrophobic compared with other protein-protein binding surfaces. Instead, the residues on the antigenic epitopes are more diverse than regular surface residues due to the evolution pressure from the host immune system. Therefore, we conclude that the general binding site prediction methods are not suitable for antigenic epitope prediction. Any future developed epitope prediction method is not recommended to claim performance improvement by comparing with binding site prediction methods.

**Table 2:** List of the protein binding site prediction methods and their obtained AUC results.

Method	URL of web server	AUC
ProMate [26]	http://bioinfo.weizmann.ac.il/promate/	0.530
ConSurf [27]	http://consurf.tau.ac.il/index_proteins.php	0.460^a^
PINUP [28]	http://sysbio.unl.edu/services/PINUP	0.562
PIER [29]	http://abagyan.ucsd.edu/PIER/pier.cgi?act=dataset	0.537

a Conserved residues are selected as for common binding site prediction.

## Discussion

Currently, various sets of attributes and classifiers have been applied by different existing epitope prediction algorithms, which naturally leads to one question: Which combination of attributes is optimal for the prediction? To answer this question, one may systematically evaluate different machine-learning algorithms on all non-redundant attributes and allocate the optimal set among them. Also of great importance to the epitope prediction research is the growth of the training data, especially the antigens that have both bounded and unbounded structures. In addition, it is also important to collect high quality independent testing data, such as the ones compiled by Liang *et al.* [22] that contain experimentally measured epitopic residues but no complex structures. We also recommend that all future researchers implement their developed algorithms as free accessible web servers or downloadable software packages, because B-cell epitope prediction algorithms will likely become more and more complicated and meta-methods usually have better prediction accuracy than any of the single algorithms (Table 1).

### Conclusions

In recent years, there have been developed a number of new conformational B-cell epitope prediction algorithms. While the prediction performance has accumulated some improvement, it is still far from satisfactory. Compared with other bioinformatic problems, antigenic epitope prediction is especially difficult due to the lack of properties that are universally observed for the antigenic epitopes but not for other protein surfaces. Additionally, common binding site prediction methods are not suitable for antigenic epitope prediction because they focus on the conservation of surface residues.

## References

[1] Parker JM, Guo D, Hodges RS (1986) New hydrophilicity scale derived from high-performance liquid chromatography peptide retention data: correlation of predicted surface residues with antigenicity and X-ray-derived accessible sites. Biochemistry 25: 5425–5432. 2430611 10.1021/bi00367a013

[2] Emini EA, Hughes JV, Perlow DS, Boger J (1985) Induction of hepatitis A virus-neutralizing antibody by a virus-specific synthetic peptide. J Virol 55: 836–839. 2991600 10.1128/jvi.55.3.836-839.1985 PMC255070

[3] Karplus PA, Schulz GE (1985) Prediction of Chain Flexibility in Proteins - a Tool for the Selection of Peptide Antigens. Naturwissenschaften 72: 212–213.

[4] Kolaskar AS, Tongaonkar PC (1990) A semi-empirical method for prediction of antigenic determinants on protein antigens. FEBS Lett 276: 172–174. 1702393 10.1016/0014-5793(90)80535-q

[5] Larsen JE, Lund O, Nielsen M (2006) Improved method for predicting linear B-cell epitopes. Immunome Res 2: 2. 16635264 10.1186/1745-7580-2-2 PMC1479323

[6] Saha S, Raghava GP (2006) Prediction of continuous B-cell epitopes in an antigen using recurrent neural network. Proteins 65: 40–48. 16894596 10.1002/prot.21078

[7] Chen J, Liu H, Yang J, Chou KC (2007) Prediction of linear B-cell epitopes using amino acid pair antigenicity scale. Amino Acids 33: 423–428. 17252308 10.1007/s00726-006-0485-9

[8] El-Manzalawy Y, Dobbs D, Honavar V (2008) Predicting linear B-cell epitopes using string kernels. J Mol Recognit 21: 243–255. 18496882 10.1002/jmr.893 PMC2683948

[9] Blythe MJ, Flower DR (2005) Benchmarking B cell epitope prediction: underperformance of existing methods. Protein Sci 14: 246–248. 15576553 10.1110/ps.041059505 PMC2253337

[10] Greenbaum JA, Andersen PH, Blythe M, Bui HH, Cachau RE, et al (2007) Towards a consensus on datasets and evaluation metrics for developing B-cell epitope prediction tools. J Mol Recognit 20: 75–82. 17205610 10.1002/jmr.815

[11] Sweredoski MJ, Baldi P (2009) COBEpro: a novel system for predicting continuous B-cell epitopes. Protein Eng Des Sel 22: 113–120. 19074155 10.1093/protein/gzn075 PMC2644406

[12] Yang X, Yu X (2009) An introduction to epitope prediction methods and software. Rev Med Virol 19: 77–96. 19101924 10.1002/rmv.602

[13] Van Regenmortel MHV (1996) Mapping Epitope Structure and Activity: From One-Dimensional Prediction to Four-Dimensional Description of Antigenic Specificity. Methods 9: 465–472. 8812702 10.1006/meth.1996.0054

[14] Kulkarni-Kale U, Bhosle S, Kolaskar AS (2005) CEP: a conformational epitope prediction server. Nucleic Acids Res 33: W168–171. 15980448 10.1093/nar/gki460 PMC1160221

[15] Andersen PH, Nielsen M, Lund O (2006) Prediction of residues in discontinuous B-cell epitopes using protein 3D structures. Protein Science 15: 2558–2567. 17001032 10.1110/ps.062405906 PMC2242418

[16] Sweredoski MJ, Baldi P (2008) PEPITO: improved discontinuous B-cell epitope prediction using multiple distance thresholds and half sphere exposure. Bioinformatics 24: 1459–1460. 18443018 10.1093/bioinformatics/btn199

[17] Ponomarenko J, Bui HH, Li W, Fusseder N, Bourne PE, et al (2008) ElliPro: a new structure-based tool for the prediction of antibody epitopes. BMC Bioinformatics 9: 514. 19055730 10.1186/1471-2105-9-514 PMC2607291

[18] Sun J, Wu D, Xu T, Wang X, Xu X, et al (2009) SEPPA: a computational server for spatial epitope prediction of protein antigens. Nucleic Acids Res 37: W612–616. 19465377 10.1093/nar/gkp417 PMC2703964

[19] Rubinstein ND, Mayrose I, Pupko T (2009) A machine-learning approach for predicting B-cell epitopes. Mol Immunol 46: 840–847. 18947876 10.1016/j.molimm.2008.09.009

[20] Rubinstein ND, Mayrose I, Martz E, Pupko T (2009) Epitopia: a web-server for predicting B-cell epitopes. BMC Bioinformatics 10: 287. 19751513 10.1186/1471-2105-10-287 PMC2751785

[21] Liang S, Zheng D, Zhang C, Zacharias M (2009) Prediction of antigenic epitopes on protein surfaces by consensus scoring. BMC bioinformatics 10: 302. 19772615 10.1186/1471-2105-10-302 PMC2761409

[22] Liang S, Zheng D, Standley DM, Yao B, Zacharias M, et al (2010) EPSVR and EPMeta: prediction of antigenic epitopes using support vector regression and multiple server results. BMC bioinformatics 11: 381. 20637083 10.1186/1471-2105-11-381 PMC2910724

[23] Zhang W, Xiong Y, Zhao M, Zou H, Ye X, et al (2011) Prediction of conformational B-cell epitopes from 3D structures by random forests with a distance-based feature. BMC bioinformatics 12: 341. 21846404 10.1186/1471-2105-12-341 PMC3228550

[24] Ponomarenko JV, Bourne PE (2007) Antibody-protein interactions: benchmark datasets and prediction tools evaluation. BMC Struct Biol 7: 64. 17910770 10.1186/1472-6807-7-64 PMC2174481

[25] El-Manzalawy Y, Honavar V (2010) Recent advances in B-cell epitope prediction methods. Immunome research 6 Suppl 2: S2. 10.1186/1745-7580-6-S2-S2 PMC2981878 21067544

[26] Neuvirth H, Raz R, Schreiber G (2004) ProMate: a structure based prediction program to identify the location of protein-protein binding sites. Journal of molecular biology 338: 181–199. 15050833 10.1016/j.jmb.2004.02.040

[27] Ashkenazy H, Erez E, Martz E, Pupko T, Ben-Tal N (2010) ConSurf 2010: calculating evolutionary conservation in sequence and structure of proteins and nucleic acids. Nucleic acids research 38: W529–533. 20478830 10.1093/nar/gkq399 PMC2896094

[28] Liang S, Zhang C, Liu S, Zhou Y (2006) Protein binding site prediction using an empirical scoring function. Nucleic acids research 34: 3698–3707. 16893954 10.1093/nar/gkl454 PMC1540721

[29] Kufareva I, Budagyan L, Raush E, Totrov M, Abagyan R (2007) PIER: protein interface recognition for structural proteomics. Proteins 67: 400–417. 17299750 10.1002/prot.21233

